# Sarmentosin alleviates doxorubicin-induced cardiotoxicity and ferroptosis *via* the p62-Keap1-Nrf2 pathway

**DOI:** 10.1080/13510002.2024.2392329

**Published:** 2024-08-16

**Authors:** Zhihui Lin, Chang Wu, Dongyan Song, Chenxi Zhu, Bosen Wu, Jie Wang, Yangjing Xue

**Affiliations:** a Department of Cardiology, The Second Affiliated Hospital and Yuying Children’s Hospital, Wenzhou Medical University, Wenzhou, People’s Republic of China; b Department of Endocrinology, The Second Affiliated Hospital and Yuying Children’s Hospital, Wenzhou Medical University, Wenzhou, People’s Republic of China

**Keywords:** Sarmentosin, Doxorubicin, cardiotoxicity, autophagy, ferroptosis

## Abstract

Doxorubicin (Dox) is extensively used as an antitumor agent, but its severe cardiotoxicity significantly limits its clinical use. Current treatments for Dox-induced cardiotoxicity are inadequate, necessitating alternative solutions. This study evaluated the effects of sarmentosin, a compound from Sedum sarmentosum, on Dox-induced cardiotoxicity and dysfunction. Sarmentosin was administered as a pretreatment to both mice and H9c2 cells before Dox exposure. Subsequently, markers of Dox-induced cardiotoxicity and ferroptosis in serum and cell supernatants were measured. Western blot analysis was utilized to detect levels of ferroptosis, oxidative stress, and autophagy proteins. Additionally, echocardiography, hematoxylin–eosin staining, ROS detection, and immunofluorescence techniques were employed to support our findings. Results demonstrated that sarmentosin significantly inhibited iron accumulation, lipid peroxidation, and oxidative stress, thereby reducing Dox-induced ferroptosis and cardiotoxicity in C57BL/6 mice and H9c2 cells. The mechanism involved the activation of autophagy and the Nrf2 signaling pathway. These findings suggest that sarmentosin may prevent Dox-induced cardiotoxicity by mitigating ferroptosis. The study underscores the potential of compounds like sarmentosin in treating Dox-induced cardiotoxicity.

## 1. Introduction

Doxorubicin (Dox), a widely utilized antineoplastic drug, is highly effective against various cancers, including breast cancer, lymphoma, and leukemia, due to its potent anti-tumor properties. However, its clinical use is often limited by significant cardiotoxicity, leading to heart failure and other cardiac complications [1,2]. Current treatments primarily manage cardiotoxicity symptoms rather than addressing the root causes. Given the substantial patient population affected by this condition, a deeper understanding of the molecular mechanisms involved is crucial. This knowledge could identify novel therapeutic targets, potentially leading to more effective treatments for Dox-induced cardiotoxicity and improving patient outcomes.

Dox-induced cardiotoxicity is a complex process involving multiple mechanisms [3–5], such as lipid peroxidation [6], mitochondrial injury [7], DNA damage [8], autophagy [9], and apoptosis [10–12]. One emerging non-apoptotic cell death mechanism implicated in Dox-induced cardiotoxicity is ferroptosis, characterized by increased lipid peroxidation due to iron-mediated lipoxygenase activation [6,13,14]. The balance between reactive oxygen species (ROS) generation and antioxidant production is vital in ferroptosis [15]. The Fenton reaction, through the Dox-Fe^2+^ complex, produces OH•, triggering lipid peroxidation and damaging cardiomyocytes *via* ferroptosis [16,17]. Furthermore, Dox downregulates Glutathione peroxidase 4 (GPX4), a pivotal anti-ferroptosis protein, in cell membranes and mitochondria [18]. This reduction, along with the inhibition of the cystine-glutamate transporter XC, induces ferroptosis. There is a significant link between ferroptosis and the Nrf2 signaling pathway [19]. Recent studies suggest that ferroptosis, through the Nrf2 pathway, significantly contributes to Dox-induced cardiotoxicity. Additionally, ferroptosis inhibitors like Fer-1 and Dexrazoxane have shown effectiveness in preventing Dox-induced cardiomyopathy. Based on these findings, a novel therapeutic strategy for the treatment of Dox-induced cardiotoxicity may be developed focusing on reducing ferroptosis.

Sarmentosin, an active compound from the herb *Sedum sarmentosum* [20,21]. exhibits several pharmacological effects, including anti-inflammatory, antifibrotic, and anti-tumor properties [22–24]. Research indicates that sarmentosin protects the cardiovascular system and possesses antioxidant activity. Its protective effects against Dox-induced cell death have been demonstrated in H9c2 cardiomyocytes and C57BL/6 mice, suggesting its potential in treating Dox-induced cardiomyopathy. Sarmentosin was found to effectively inhibit iron accumulation, lipid peroxidation, and oxidative stress, thereby mitigating Dox-induced ferroptosis and cardiotoxicity in both C57BL/6 mice and H9c2 cells. The underlying mechanism involves the promotion of autophagy and the Nrf2 signaling pathway. Thus, sarmentosin appears to protect against Dox-induced cardiotoxicity by alleviating ferroptosis.

## 2. Materials and methods

### 2.1. Animals

This study was reviewed and approved by the Wenzhou Medical University's Animal Care and Use Committee (wydw2023-0245). Male C57BL/6 mice, 6–8 weeks old, were provided by the Zhejiang Vital River Laboratory Animal Technology Co, Ltd.

### 2.2. Experimental design

A random sample of mice was divided into three groups: (1) Normal control group, receiving vehicle (saline); (2) Dox group, receiving saline intragastrically for 3 weeks, followed by six intraperitoneal injections of Dox (15 mg/kg cumulative dose) over 2 weeks starting one week after saline administration; (3) Dox + Sarmentosin group, receiving sarmentosin (20 mg/kg/day) intragastrically for 3 weeks, with six intraperitoneal injections of Dox over 2 weeks starting one week after the first sarmentosin administration.

*In vitro*, H9c2 cells were pretreated with 10 µM chloroquine (CQ) for 12 hours, followed by co-treatment with 10 µM CQ and 10 µM sarmentosin for another 12 hours. The medium was then replaced, and cells were treated with 1 µM Dox and 10 µM sarmentosin for 24 hours.

### 2.3. Assessment of cardiac structure and function

Echocardiography was performed using an ultrasound machine (ACUSON Sequoia 512, SIEMENS Corporation) under inhalation anesthesia with 2% isoflurane. M-mode echocardiograms were obtained at the papillary muscle level from parasternal long axes. Measurements included LVESD, LVEDD, LVSV, and LVDV. Left ventricular ejection fraction (LVEF) and left ventricular fractional shortening (LVFS) were calculated using the formulas: LVEF = (LVDV−LVSV)/LVDV × 100%; LVFS = (LVDD− LVSD)/LVDD × 100%. The mean value was calculated from three measurements [25]. Serum, supernatants, and proteins were separated following treatments, and various indicators were evaluated using biochemical kits from Nanjing Jiancheng. These indicators included lactate dehydrogenase (LDH), creatine kinase MB isoenzyme (CK-MB), malondialdehyde (MDA), reduced glutathione (GSH), and superoxide dismutase (SOD). Cardiac troponin T (cTnT) levels were determined using ELISA kits (E-ELM1801c, Elabscience).

### 2.4. Histopathologic assay

Tissues were embedded in paraffin, fixed in formalin, and stained with hematoxylin–eosin (HE) for histological examination using a light microscope (Olympus BX51).

### 2.6. Cell viability assay

Cells were plated in 96-well plates at a density of 5 × 10^3^ cells per well. Post-treatment with Dox and sarmentosin, cell viability was assessed using Cell Counting Kit-8 (CCK-8) (#C0038, Beyotime) for 2 hours in the dark.

### 2.7. Iron assay

Intracellular ferrous iron levels were measured following the manufacturer’s instructions with an iron assay kit (#ab83366, Abcam).

### 2.8. Lipid ROS detection

Following Dox and sarmentosin treatment, cells were washed three times with sterile PBS after removing the supernatant. BODIPY 581/591 C11 (#D3861, Thermo) working solution was added, and cells were incubated for 30 minutes, followed by three sterile PBS washes.

### 2.9. Ad-mCherry-GFP-LC3B transfection assay

Transfection with Ad-mCherry-GFP-LC3B adenovirus was performed according to the manufacturer's protocol (#C3011-10 ml, Beyotime), with cells grown in 6-well plates overnight and transfected for 24 hours before sarmentosin pretreatment.

### 2.10. Cellular immunofluorescence

H9c2 cells were fixed with 4% paraformaldehyde for 10 minutes, permeabilized with 0.3% Triton for 10 minutes, blocked with 5% BSA for 2 hours, and incubated overnight at 4°C with primary antibodies. Secondary antibody incubation was conducted for 1 hour in the dark, followed by DAPI staining. Immunofluorescence quantification was performed using Image J.

### 2.11. Transient transfection of siRNA

The sense sequence for the Nrf2 small interference RNA (siRNA) was 5′ – GGGUAAGUCGAGAAGUGUUTT-3′, and the antisense sequence was 5′ – AACACUUCUCGACUUACCCTT-3′. H9c2 cells were transfected with siRNA using Lipofectamine^TM^ 2000 reagent (Invitrogen, Carlsbad).

### 2.12. Co-immunoprecipitation

Cell lysates were centrifuged for 10 minutes at 12,000 rpm, then incubated with the primary antibody of the target protein for 12 hours at 4°C. Agarose beads were then added, and after 4 hours, the beads were washed three times and mixed with SDS for Western blot analysis using absin reagent (#abs955, absin).

### 2.13. Western blot analysis

Lysates from tissue or cells were prepared with RIPA lysis buffer (#P0013B, Beyotime). Protein concentration was determined using the BCA kit (#P0012, Beyotime). Proteins were separated on polyacrylamide gels based on their molecular weight and transferred to PVDF membranes (#ISEQ85R, Millipore Corporation). Membranes were blocked with 5% skim milk for 2 hours at 4°C, followed by overnight incubation with the primary antibody. The next day, membranes were incubated with goat anti-rabbit IgG (#bl003a, Biosharp). Using the ChemiDocTM XRS + system and the Image Lab software (Bio-Rad), target protein signals were visualized. Using Image J to detected signal intensity of the protein.

### 2.14. Reagents and antibodies

**Figure 1.**
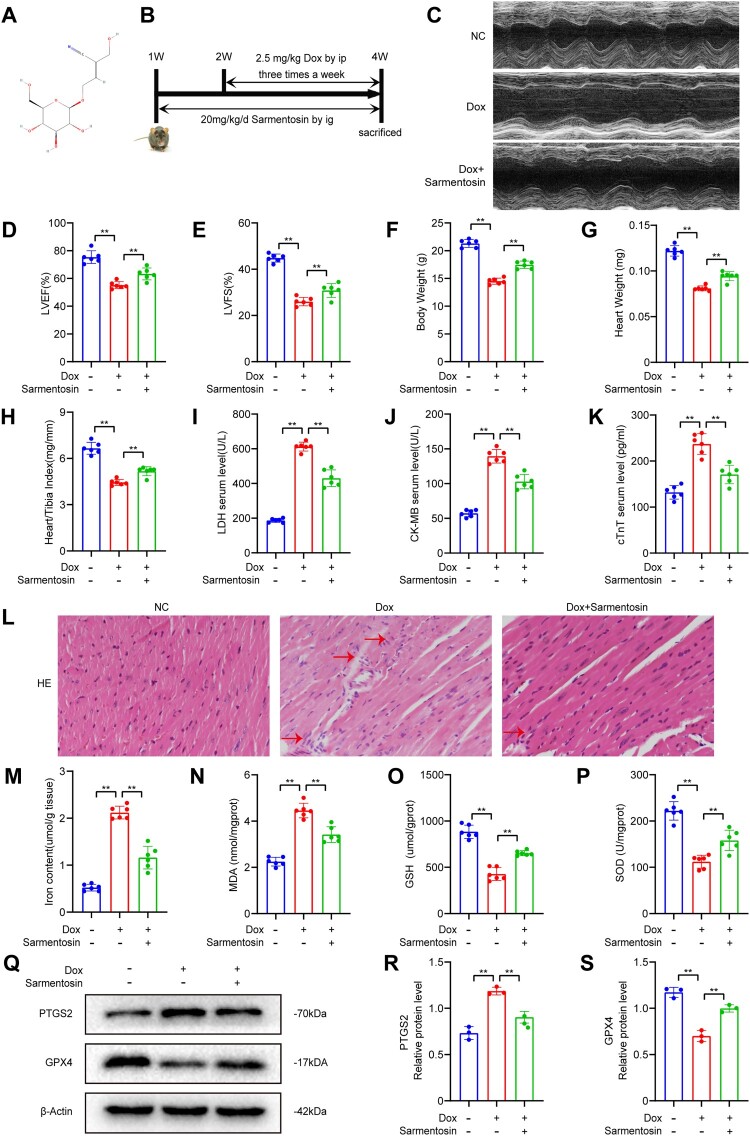
Cardiotoxicity and ferroptosis caused by Dox were alleviated by Sarmentosin *in vivo*. (A) Chemical structure of sarmentosin. (B) Establishment of the Dox model. (C) Representative echocardiogram images of each group. (D) LVEF, n = 6. (E) LVFS, n = 6. (F-H) Weight of the body, heart weight, and index of heart/tibia, n = 6. (I-K) Serum levels of LDH, CKMB, and cTnT in mice, n = 6. (L) The staining of HE in representative images (Scale bar: 50 µm), n = 6. (M-P) Myocardial iron content, MDA level, GSH level, and SOD level, n = 6. (Q) Assayed with Western blotting *in vivo* for PTGS2 and GPX4. (R-S) Expression of PTGS2 and GPX4 proteins quantitatively analyzed. A comparison was made between levels of normalized expression and levels of β-actin, n = 3. Data are means ± SD, *P < 0.05; ** *P* < 0.01.

Sarmentosin (C_11_H_17_NO_7_, purity ≥ 98%, Figure 1A) was procured from ChemFaces Biochemical Co., Ltd (China). Doxorubicin (#D1515) and CQ (#C6628) were obtained from Sigma-Aldrich. Primary antibodies included PTGS2 (#A1253, ABclonal), GPX4 (#A11243, ABclonal), Nrf2 (#12721, CST), HO-1 (#10701- 1 – AP, Proteintech), NQO1 (#A23486, ABclonal), ATG5 (#12994, CST), Keap1 (#10503-2-AP, proteintech), P62 (#23214, CST), LC3B (#A19665, ABclonal), and β-actin (#AC026, ABclonal).

### 2.15. Statistical analysis

Using SPSS 22.0, the data was analyzed and presented as mean + standard deviation (SD). All of the data we collected had a normal distribution. A two-tail unpaired Student's t-test was used to compare the two experimental groups. For comparisons of more than two groups, one-way analysis of variance followed by Duncan's T3 multiple-range test was used. *P *< 0.05 was considered statistically significant.

## 3. Results

### 3.1. Sarmentosin alleviates Dox-induced cardiotoxicity and ferroptosis in vivo

The chemical structure of sarmentosin is depicted in Figure 1A. A mouse model was established to investigate cardiotoxicity induced by Dox treatment and to confirm the protective effects of sarmentosin (Figure 1B). Several parameters, including body weight, heart weight, heart weight/tibial length ratio, LDH, CK-MB, and cTnT, were measured in a preliminary experiment with different concentrations of sarmentosin (10, 20, 40 mg/kg/d) to assess its effects on Dox-induced cardiotoxicity (Supplementary Fig. 1A-F). The optimal *in vivo* concentration of sarmentosin was determined to be 20 mg/kg, which was used for subsequent *in vivo* experiments. Additionally, sarmentosin alone was tested and found to neither damage the heart nor affect iron content and MDA levels in mice cardiac tissues (Supplementary Fig. 1G-J).

Cardiac function in each group was evaluated using echocardiography (Figure 1C). The Dox group exhibited significantly lower LVEF and LVFS compared to the control group, indicating severe cardiac dysfunction and confirming the establishment of the cardiotoxicity model. In the Dox + Sarmentosin group, LVEF and LVFS were significantly improved compared to the Dox group (Figure 1D-E), demonstrating that sarmentosin pretreatment mitigated cardiotoxicity. The Dox-induced cardiotoxicity in mice manifested as reduced body weight, heart weight, and heart weight/tibial length, and sarmentosin alleviated these changes (Figure 1 F-H). Sarmentosin also significantly decreased LDH, CK-MB, and cTnT levels (Figure 1 I-K) and alleviated Dox-induced histopathological damage to mice cardiac tissues, as observed in HE-stained tissues (Figure 1L).

To investigate the effect of sarmentosin on Dox-induced ferroptosis, ferroptosis and oxidative stress levels were measured post-treatment. Sarmentosin significantly inhibited the Dox-induced increase in iron levels and MDA, a byproduct of lipid peroxidation (Figure 1M-N). Additionally, sarmentosin prevented the Dox-induced decrease in GSH and SOD production in cardiac tissue (Figure 1O-P). Western blot analysis revealed that sarmentosin modulated ferroptosis-related protein expression in myocardial tissue (Figure 1Q), preventing changes associated with Dox-induced ferroptosis (Figure 1R-S). These findings suggest that sarmentosin protects against Dox-induced ferroptosis *in vivo* by reducing oxidative stress and modulating the expression of ferroptosis-related proteins.

### 3.2. Sarmentosin alleviated Dox-induced cardiotoxicity and ferroptosis *in vitro*

**Figure 2.**
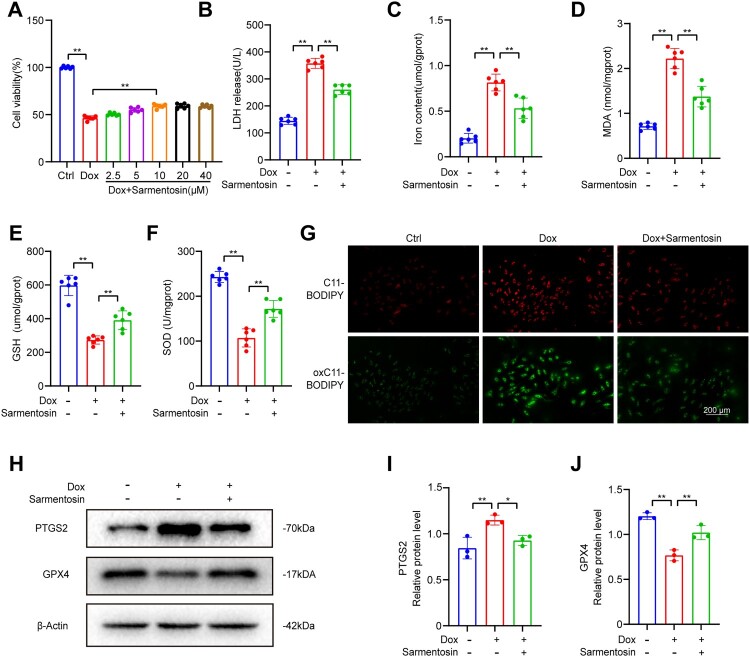
Cardiotoxicity and ferroptosis caused by Dox were alleviated by Sarmentosin *in vitro*. (A) Each group was tested for cell viability, n = 6. (B) *In vitro* levels of LDH, n = 6. (C-F) In H9c2 cells, the iron content, MDA level, GSH level, and SOD level were determined, n = 6. (G) C11-BODIPY fluorescence of lipid ROS in each group (Scale bar: 200 µm), n = 3. (H) Assayed with Western blotting *in vitro* for PTGS2 and GPX4. (I-J) Expression of PTGS2 and GPX4 proteins quantitatively analyzed. A comparison was made between levels of normalized expression and levels of β-actin, n = 3. Data are means ± SD, *P < 0.05; ** *P* < 0.01.

Firstly, we determined half maximum excitation concentration (EC50) values of sarmentosin to be 400 µM (Supplementary Fig. 2A). In experiments with H9c2 cells challenged by Dox, sarmentosin effectively reduced Dox-induced injury at a concentration of 10 µM (Figure 2A). Consequently, this concentration was used in subsequent cell experiments. Additionally, treatment with sarmentosin alone did not affect LDH levels, iron content, or MDA levels *in vitro* (Supplementary Fig. 2B-D).

Sarmentosin treatment reduced Dox-mediated elevation of LDH levels in cell supernatants (Figure 2B). The levels of ferroptosis and oxidative stress in H9c2 cells were evaluated to determine whether sarmentosin affects ferroptosis induced by Dox. We found that sarmentosin significantly reduced iron levels and MDA (Figure 2C, D) and elevated GSH and SOD in H9c2 cells (Figure 2E, F). Furthermore, sarmentosin suppressed lipid ROS generation (Figure 2G), as indicated by reduced levels of oxC11-BODIPY (oxidized) compared to C11-BODIPY (unoxidized). To better evaluate ferroptosis levels in H9c2 cells, we determined the levels of ferroptosis-related proteins using western blotting (Figure 2H). We observed that sarmentosin suppressed the Dox-mediated increase in PTGS2 expression and promoted GPX4 expression in H9c2 cells (Figure 2I, J). These results suggest that sarmentosin protected cardiomyocytes by suppressing ferroptosis.

### 3.3. Sarmentosin altered the Nrf2 signal pathway

**Figure 3.**
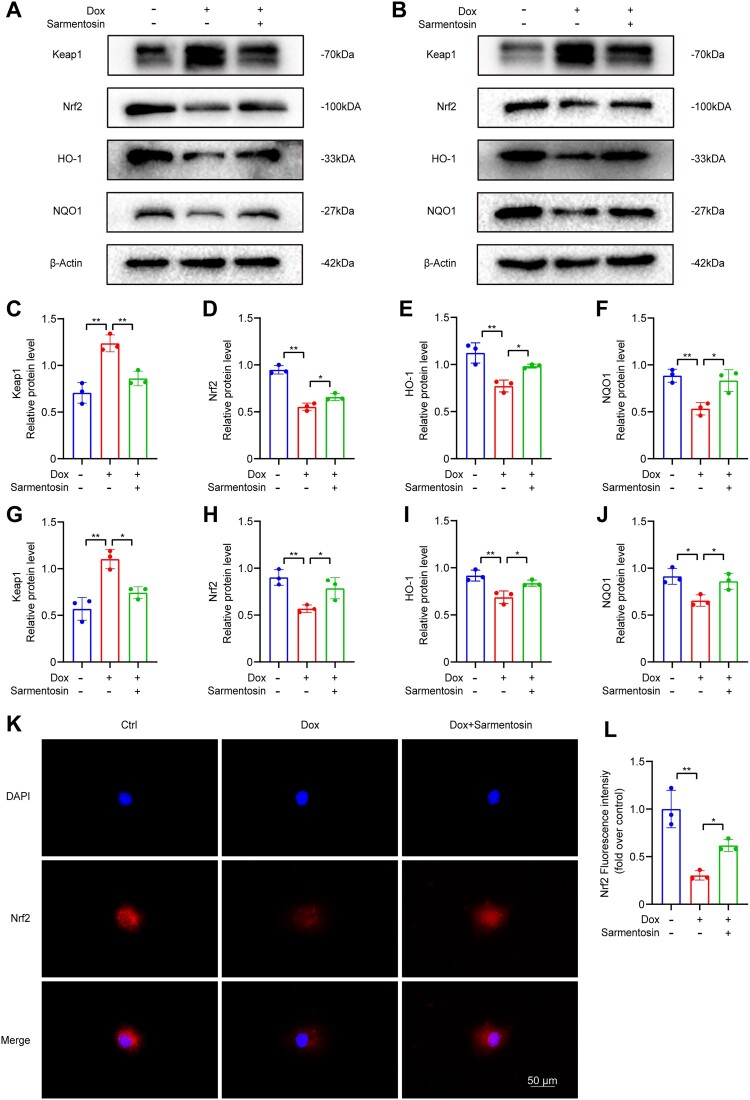
Sarmentosin adjusted Nrf2 signal pathway *in vivo* and *in vitro*. (A) Assayed with Western blotting *in vivo* for Keap1, Nrf2, HO-1 and NQO1. (B) Assayed with Western blotting *in vitro* for Keap1, Nrf2, HO-1 and NQO1. (C-F) Expression of Keap1, Nrf2, HO-1 and NQO1 proteins quantitatively analyzed *in vivo*. A comparison was made between levels of normalized expression and levels of β-actin n = 3. (G-J) Expression of Keap1, Nrf2, HO-1 and NQO1 proteins quantitatively analyzed *in vitro*. A comparison was made between levels of normalized expression and levels of β-actin, n = 3. (K) Immunofluorescence staining of Nrf2 in each group (Scale bar: 50 µm), n = 3. (L) Quantification of Nrf2 relative fluorescence intensity. Data are means ± SD, *P < 0.05; ** *P* < 0.01.

Given the close relationship between ferroptosis and Nrf2 signaling, changes in Nrf2 signaling were analyzed *via* Western blot after sarmentosin treatment in myocardial tissue (Figure 3A). Dox treatment increased Keap1 levels and significantly reduced Nrf2, HO-1, and NQO1 expression *in vivo*. However, sarmentosin treatment significantly decreased Keap1 levels and increased the expression of Nrf2, HO-1, and NQO1 (Figure 3C-F).

In addition, the expression levels of Keap1, Nrf2, HO-1 and NQO1 were also assessed in H9c2 cells by western blotting (Figure 3B) and revealed that the protein expression of Keap1, Nrf2, HO-1 and NQO1 was similar to the result *in vitro* (Figure 3G-J). Moreover, immunofluorescence confirmed that sarmentosin increased Nrf2 expression in H9c2 cells (Figure 3K-L). According to these results, sarmentosin descreased Keap1 expression, which in turn led to increases in Nrf2, HO-1 and NQO1 expression downstream.

### 3.4. Sarmentosin alleviated Dox-induced cardiotoxicity and ferroptosis by activating the Nrf2 signaling pathway

**Figure 4.**
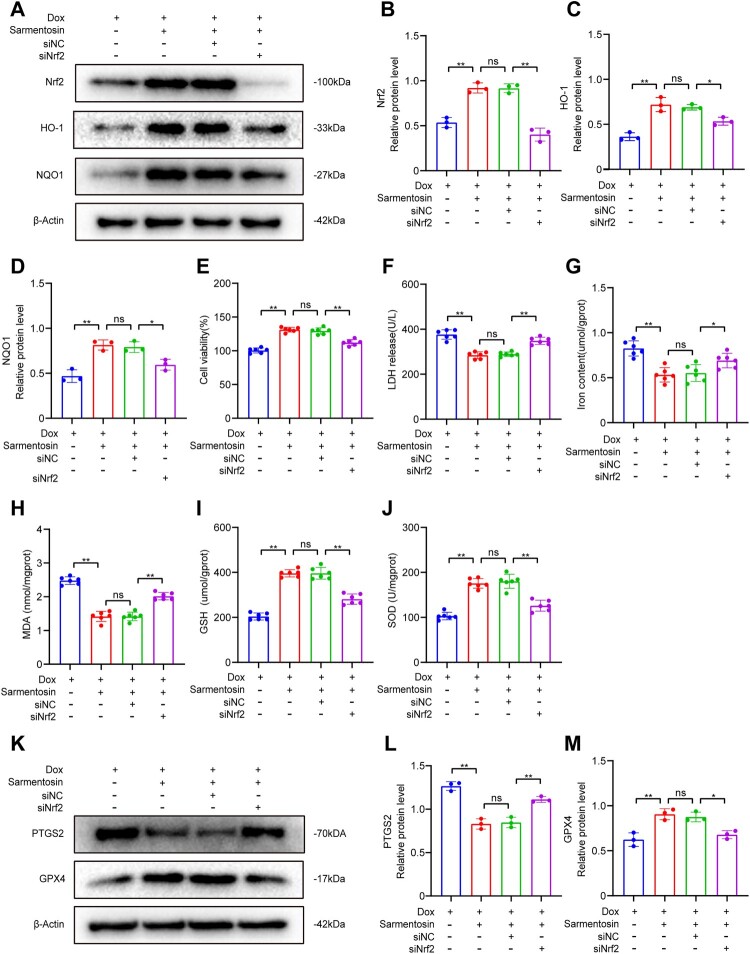
Sarmentosin alleviated doxorubicin-induced cardiotoxicity and ferroptosis *via* a Nrf2-dependent mechanism. (A) Assayed with Western blotting *in vitro* for Nrf2, HO-1 and NQO1. (B-D) Expression of Nrf2, HO-1 and NQO1 proteins quantitatively analyzed. A comparison was made between levels of normalized expression and levels of β-actin, n = 3. (E) Each group was tested for cell viability, n = 6. (F) *In vitro* levels of LDH, n = 6. (G-J) In H9c2 cells, the iron content, MDA level, GSH level, and SOD level were determined, n = 6. (K) Assayed with Western blotting *in vitro* for PTGS2 and GPX4. (L-M) Expression of PTGS2 and GPX4 proteins quantitatively analyzed. A comparison was made between levels of normalized expression and levels of β-actin, n = 3. Data are means ± SD, *P < 0.05; ** *P* < 0.01.

To elucidate the mechanism underlying sarmentosin's protective effects, the role of Nrf2 signaling in inhibiting ferroptosis was investigated. Nrf2, a transcription factor regulating antioxidant and detoxification genes, is essential in protecting cells from oxidative stress. Western blot analysis of H9c2 cells treated with siNrf2 revealed altered Nrf2 levels (Supplementary Fig. 2E-F). Additionally, siNrf2 significantly reduced the proteins expression of Nrf2, HO-1 and NQO1, reversing the effects of sarmentosin (Figure 4A-D).

Similarly, with siNrf2, a decrease in cell viability, LDH, GSH and SOD levels was observed, as well as an increase in iron and MDA levels (Figure 4 E-J). Following Dox-induced injury, siNrf2 treatment increased PTGS2 expression and decreased GPX4 expression, which was the opposite of what was observed when sarmentosin was applied. (Figure 4 K-M). These results suggest that sarmentosin's protective effects against Dox-induced cardiotoxicity and ferroptosis are mediated, at least in part, by the activation of Nrf2 signaling.

### 3.5. Sarmentosin treatment promoted autophagic flux in models of Dox-induced cardiotoxicity

**Figure 5.**
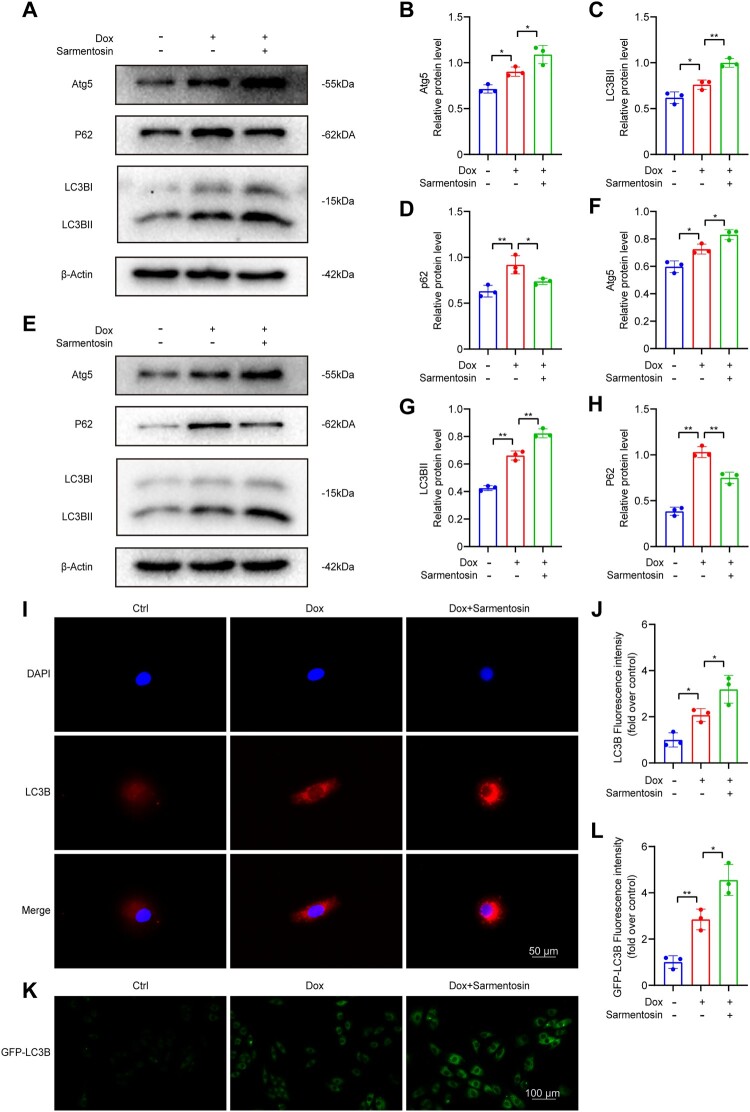
Sarmentosin treatment promoted autophagic flux in models of Dox-induced cardiotoxicity. (A) Assayed with Western blotting *in vivo* for Atg5, LC3B and P62. (B-D) Expression of Atg5, LC3B and P62 proteins quantitatively analyzed *in vivo*. A comparison was made between levels of normalized expression and levels of β-actin, n = 3. (E) Assayed with Western blotting *in vitro* for Atg5, LC3B and P62. (F-H) Expression of Atg5, LC3B and P62 proteins quantitatively analyzed *in vitro*. A comparison was made between levels of normalized expression and levels of β-actin, n = 3. (I) Immunofluorescence staining of LC3B in each group (Scale bar: 50 µm), n = 3. (J) Quantification of LC3B relative fluorescence intensity. (K) Representative images of H9c2 cells transfected Ad-mCherry-GFP-LC3B adenovirus in each group (Scale bar: 100 µm), n = 3. (L) Quantification of GFP-LC3B relative fluorescence intensity. Data are means ± SD, *P < 0.05; ** *P* < 0.01.

Autophagy plays a significant role in Dox-induced cardiotoxicity, as shown in several studies. To explore this, the impact of sarmentosin on autophagy was assessed. In mouse heart tissue, western blot analysis was used to examine the expression of autophagy-related proteins Atg5, P62, and LC3B (Figure 5A). Results showed that sarmentosin increased the protein levels of Atg5 and LC3BII and decreased P62 levels, indicating enhanced autophagosome formation and degradation (Figure 5B-D). Similarly, in H9c2 cells, sarmentosin promoted autophagosome formation and degradation, as evidenced by increased Atg5 and LC3BII levels and decreased P62 levels (Figure 5E-H). Immunofluorescence microscopy of H9c2 cells stained with LC3B confirmed sarmentosin's promotion of autophagosome generation (Figure 5I-J). Further, transfection of H9c2 cells with Ad-mCherry-GFP-LC3B adenovirus demonstrated that sarmentosin enhanced autophagosome formation (Figure 5K-L). These results suggest that sarmentosin promotes autophagic flux in models of Dox-induced cardiotoxicity.

### 3.6. Sarmentosin ameliorated Dox-induced injury and inhibited ferroptosis by promoting autophagosome degradation *in vitro*

**Figure 6.**
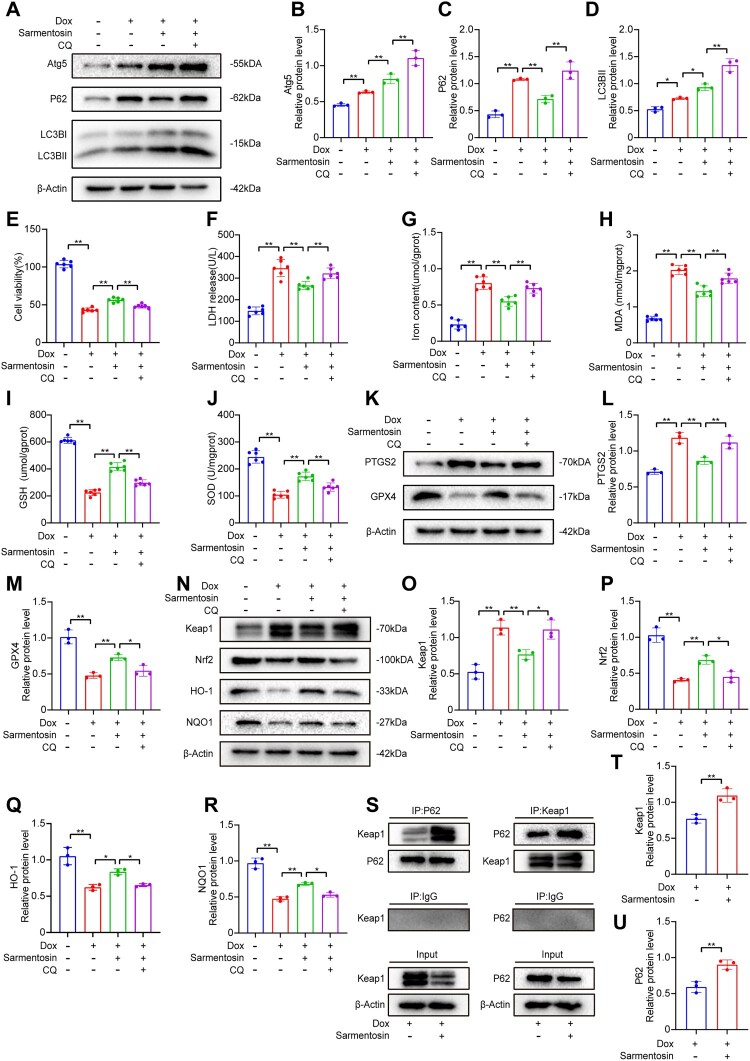
Sarmentosin ameliorated Dox-induced injury and inhibited ferroptosis by promoting autophagosome degradation in vitro. (A) Assayed with Western blotting *in vivo* for Atg5, P62 and LC3B. (B-D) Quantitative analysis of the expression of Atg5, P62 and LC3B proteins. A comparison was made between levels of normalized expression and levels of β-actin, n = 3. (E) Each group was tested for cell viability, n = 6. (F) *In vitro* levels of LDH, n = 6. (G-J) In H9c2 cells, the iron content, MDA level, GSH level, and SOD level were determined, n = 6 (K) Assayed with Western blotting *in vitro* for PTGS2 and GPX4. (L-M) Quantitative analysis of the expression of PTGS2 and GPX4 proteins. A comparison was made between levels of normalized expression and levels of β-actin, n = 3. (N) Assayed with Western blotting *in vitro* for Keap1, Nrf2, HO-1 and NQO1. (O-R) Quantitative analysis of the expression of Keap1, Nrf2, HO-1 and NQO1 proteins. A comparison was made between levels of normalized expression and levels of β-actin, n = 3. (S) Combination of Keap1 protein with P62 was identified by Co-IP. Normalized expression levels were compared to β-actin, n = 3. (T-U) Western blotting analysis of Co-IP, n = 3. Data are means ± SD, *P < 0.05; ** *P* < 0.01.

Autophagy and ferroptosis are critical in the pathogenesis of Dox-induced cardiomyopathy. To elucidate how sarmentosin mitigates Dox-induced cardiotoxicity and ferroptosis, CQ was employed to inhibit autophagosome clearance. CQ treatment significantly impeded autophagosome clearance, leading to their increased accumulation (Figure 6A-D). This intervention partially reversed the therapeutic effects of sarmentosin, increasing cytotoxicity post-Dox injury (Figure 6E). Additionally, CQ inhibited autophagy degradation, elevated LDH, iron, and MDA levels, and reduced GSH and SOD levels after Dox-induced injury (Figure 6F-J). Furthermore, CQ treatment upregulated PTGS2 expression and downregulated GPX4 expression following Dox-induced injury (Figure 6K-M). To further investigate sarmentosin's effect on autophagic flux and its impact on Dox-induced cardiotoxicity and ferroptosis, comparisons were made between the Dox + CQ group and the Dox + CQ + Sarmentosin group. The findings demonstrated that sarmentosin alleviates Dox-induced cardiotoxicity and ferroptosis by promoting autophagic flux (Supplementary Fig. 2G-N).

Interestingly, CQ treatment was found to alter Nrf2 signaling (Figure 6N). CQ reduced the protein expression of Keap1 and its downstream targets Nrf2, HO-1, and NQO1 in H9c2 cells (Figure 6O-R). Co-immunoprecipitation and western blot analysis revealed that sarmentosin enhanced the interaction between Keap1 and P62 (Figure 6S-U). These results suggest that P62 more readily binds to Keap1, facilitating Nrf2 expression in the Dox + Sarmentosin group. The removal of autophagosomes appears to eliminate Keap1, and sarmentosin accelerates Keap1 degradation through autophagy. Overall, these results suggest that sarmentosin exerts its protective effects against Dox-induced cardiomyopathy by promoting autophagy and its interplay with ferroptosis and Nrf2 signaling.

## 4. Discussion

**Figure 7.**
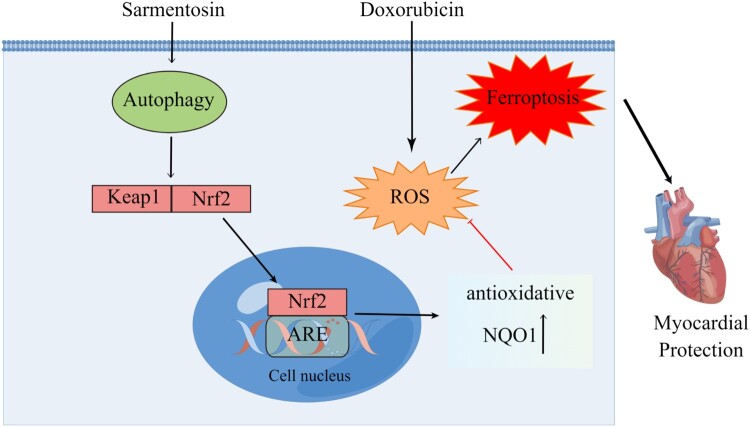
Schematic: As a result of its ability to promote autophagy and Nrf2 signaling, sarmamentosin relieves the cardiotoxicity and ferroptosis induced by doxorubicin.

The adverse cardiovascular effects of cancer treatment pose a significant concern for survivors [26,27]. Dox, a widely used chemotherapeutic agent, is notably cardiotoxic [28]. Despite efforts to mitigate this toxicity, Dox remains prevalent due to the limited availability of alternative treatments [29–32]. This study investigated the cardioprotective effects of sarmentosin against Dox-induced cardiotoxicity, marking the first exploration of sarmentosin in this context. The findings suggest that sarmentosin may offer a viable treatment option for Dox-induced cardiotoxicity by preserving cardiac structure and function Figure 7.

Mechanisms underlying Dox cardiotoxicity include apoptosis [33], autophagy [34], and necroptosis [35]. Dox-induced ferroptosis is initiated in mitochondria, where Dox increases lipid peroxides and downregulates GPX4, leading to excessive lipid peroxidation [36]. Studies have shown that ferrostatin-1, a ferroptosis inhibitor, significantly reduces mortality by inhibiting ferroptosis. Conversely, treatments with emricasan (an apoptosis inhibitor) and necrostatin-1 (a receptor-interacting protein kinase 1 inhibitor) did not provide significant protection against Dox effects [37]. Ferroptosis is characterized by severe lipid peroxidation mediated by ROS and iron accumulation [38] and involves both oxidant and antioxidant mechanisms. Ferroptotic cells typically exhibit necrosis-like changes, such as swelling, plasma membrane rupture, and mitochondrial injury, distinct from the morphological features of apoptosis [39]. Additionally, protein arginine methyltransferase 4 (PRMT4) has been found to interact with Nrf2, promoting its enzymatic methylation, reducing nuclear translocation of Nrf2, and decreasing GPX4 expression [40]. Our study demonstrates that sarmentosin protects cardiomyocytes by inhibiting ferroptosis and enhancing GPX4 expression. This catalytic activity prevents lipid peroxidation toxicity and maintains membrane homeostasis, positioning GPX4 as a key target for suppressing ferroptosis [41].

Several factors, including mitochondrial respiration, are impacted by Dox cardiotoxicity, with oxidative stress being a primary mechanism. Nrf2 transcriptional targets regulate iron metabolism, detoxification of reactive intermediates, and glutathione synthesis to mitigate oxidative stress [42]. Numerous studies have shown that upregulating the Nrf2/HO-1 signaling pathway effectively alleviates Dox-induced cardiotoxicity [43,44]. HO-1 also plays a role in iron homeostasis [45]. However, this study has limitations. The effect of sarmentosin on the relationship between HO-1 and iron homeostasis was not explored; the focus was on sarmentosin's impact on Nrf2 and ferroptosis. The findings indicate a significant reduction in Nrf2 expression in the hearts of the Dox group compared to the control group, suggesting that Nrf2 inhibition contributes to cardiac iron toxicity in Dox-treated mice. Sarmentosin treatment enhanced Nrf2 expression and related pathways, alleviating iron toxicity. In Dox-treated mice, sarmentosin reduced iron atrophy by activating the Nrf2 signaling pathway. Dox-induced cardiotoxicity is complex, with variable results regarding Nrf2 and its downstream molecules. While Nrf2's protective effects in Dox-induced cardiotoxicity are widely reported [40,46], some studies have observed negative effects [37]. This discrepancy may be due to the influence of oxidative stress on downstream Nrf2 factors. NQO1, which regulates cell cycle progression at the G2/M phase, is not active in non-replicating cardiac tissue [47], but is associated with cellular senescence [48,49], which can lead to cardiovascular disease [50]. Therefore, NQO1 may be related to the senescence of cardiac cells.

Autophagy, a process involving the degradation and recycling of cellular components in a homeostatic manner [51], occurs under normal and stress conditions [52,53]. Research on autophagy's role in Dox-induced cardiotoxicity has produced conflicting results. Some studies demonstrate a protective effect [25], while others suggest that blocking autophagy alleviates Dox-induced cytotoxicity. These differences may be due to variations in autophagy phases, Dox dosages, and durations, as well as the specifics of the interventions used [54,55]. In Dox-induced cardiotoxicity, autophagy is crucial, but its relationship with ferroptosis and Nrf2 is unclear. Keap1, a scaffold protein, facilitates Nrf2 ubiquitination and degradation in the cytosol. During oxidative stress, Keap1 is recruited from Keap1-Nrf2 by the autophagic adapter protein p62 [56]. Sarmentosin treatment was found to enhance the interaction between p62 and Keap1, accelerating Keap1 degradation.

In conclusion, sarmentosin alleviates Dox-induced cardiotoxicity by mitigating ferroptosis and promoting autophagy to modulate the Nrf2 signaling pathway.

## Supplementary Material

Supplementary Fig1.docx

Supplementary Fig2.docx

## Data Availability

All data from this study are available upon reasonable request by contacting the corresponding author.

## Abbreviations

Abbreviations: CK-MB: creatine kinase MB isoenzyme; CQ: chloroquine; Doxorubicin: Dox; GSH: reduced glutathione; LDH: lactate dehydrogenase; MDA: malondialdehyde; SOD: superoxide dismutase
