# Supplementary material for: Sarmentosin alleviates doxorubicin-induced cardiotoxicity and ferroptosis via the p62-Keap1-Nrf2 pathway
Source: Redox Rep. 2024 Aug 16;29(1):2392329. doi: 10.1080/13510002.2024.2392329 (PMC11332294; doi:10.1080/13510002.2024.2392329)
Supplement: Supplementary Fig1.docx [file YRER_A_2392329_SM9316.docx]

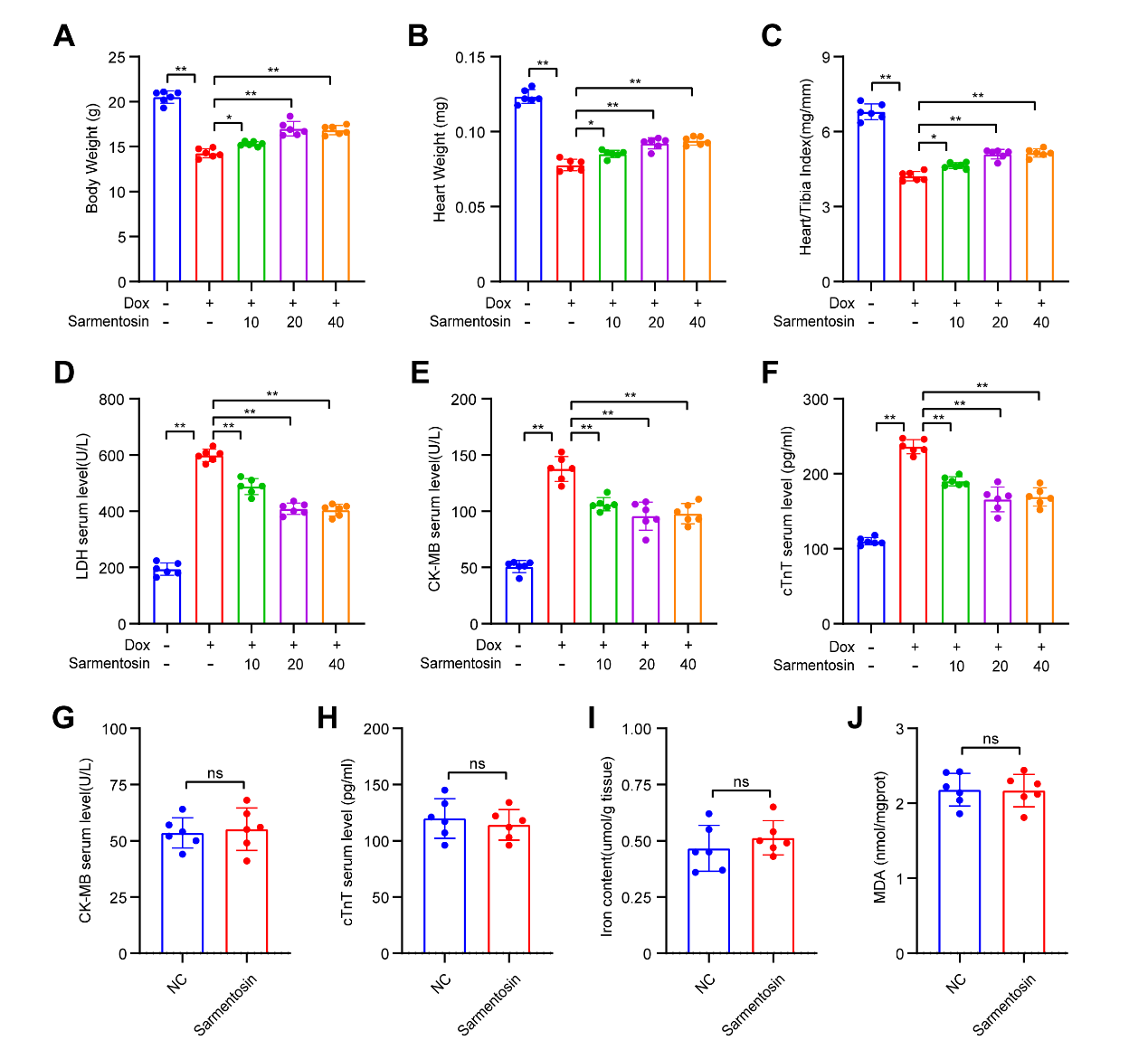


**Supplementary Fig. 1.** (A-C) Effects of different concentrations of sarmentosin (10, 20, 40 mg/kg/d) to treat Dox-induced cardiotoxicity. Body weight, heart weight and heart /Tibia index, n = 6 (D-F) Serum levels of LDH, CK- MB, and cTnT in mice, n = 6. (G-H) Serum levels of LDH, CKMB, and cTnT in mice, n = 6. (I-J) Myocardial iron content, MDA level, n = 6. Data are means ± SD, *P < 0.05; ** P < 0.01.
