# Supplementary material for: Sarmentosin alleviates doxorubicin-induced cardiotoxicity and ferroptosis via the p62-Keap1-Nrf2 pathway
Source: Redox Rep. 2024 Aug 16;29(1):2392329. doi: 10.1080/13510002.2024.2392329 (PMC11332294; doi:10.1080/13510002.2024.2392329)
Supplement: Supplementary Fig2.docx [file YRER_A_2392329_SM9315.docx]

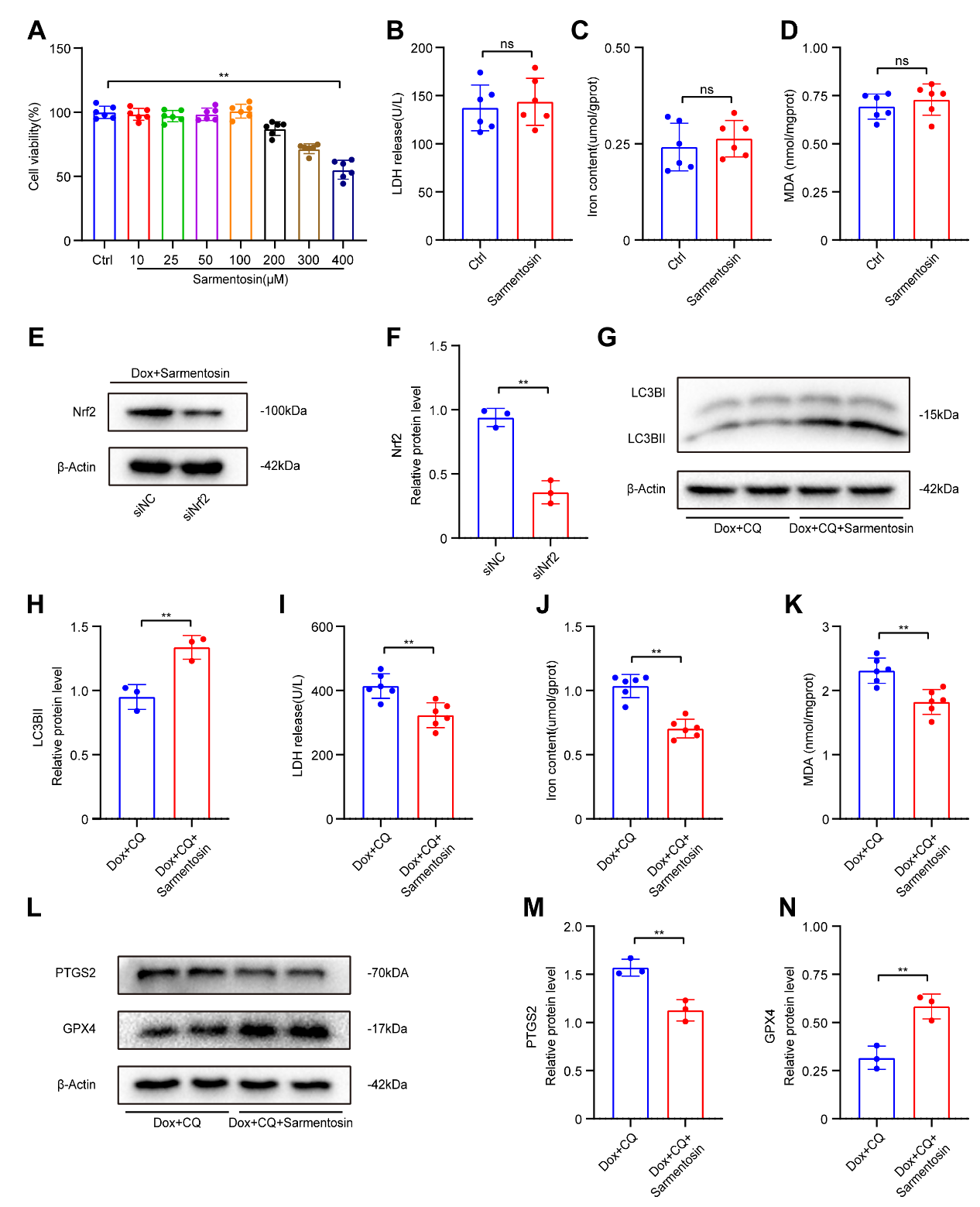


**Supplementary Fig. 2.** (A) Effects of different concentrations of sarmentosinon on H9C2 cell viability, n = 6. (B) *In vitro* levels of LDH, n = 6. (C-D) In H9C2 cells, the iron content and MDA level were determined, n = 6. (E) Assayed with Western blotting for Nrf2. (F) Expression of Nrf2 proteins quantitatively analyzed. A comparison was made between levels of normalized expression and levels of β-actin, n = 3. (G) Assayed with Western blotting for LC3B. (H) Expression of LC3B proteins quantitatively analyzed. A comparison was made between levels of normalized expression and levels of β-actin, n = 3. (I) *In vitro* levels of LDH, n = 6. (J-K) In H9C2 cells, the iron content and MDA level were determined, n = 6. (L) Assayed with Western blotting *in vitro* for PTGS2 and GPX4. (M-N) Expression of PTGS2 and GPX4 proteins quantitatively analyzed. A comparison was made between levels of normalized expression and levels of β-actin, n = 3. Data are means ± SD, *P < 0.05; ** P < 0.01.
